# Product News

**DOI:** 10.1155/S1463924680000673

**Published:** 1980


### Product News

**Editor's Note:** *The address of the manufacturer/supplier appears in italics at the end of each item. In some cases this address will be that of a subsidiary to the manufacturing company as the address given is that from which the information has been obtained.*

### Micro pH/blood gas analyser

Radiometer has introduced a programmable micro pH/blood gas analyser, the ABL3 Acid-base laboratory. It measures pH, $PCO_2$, $PO_2$, haemoglobin and barometric pressure and calculates seven additional parameters. To perform a measurement, the operator needs only to introduce the 125 μl capillary sample. All other functions, such as rinsing, calibration and recording of results, are performed automatically. A keyboard and screen (CRT) allow for communication with the built-in computer. The CRT provides information concerning the performance of the ABL3, calibration data, warning etc. The keyboard allows for requesting special programmes such as setting calibrating drift limits, entering $P_{50}$ values, or changing the frequency of the one- and/or two-point calibrations. Special programmes for soaking, adjustment of the haemoglobin or barometer channels and selection of conventional or SI units are also available.

*Radiometer A/S, 72 Emdrupvej, DK-2400 Copenhagen NV, Denmark.*

### Isotope mass spectrometer

The mass spectrometer MAT 261 is a computer controlled instrument for the determination of isotope abundances in solid samples using thermal ionisation. It has an automatic turret-type magazine for 13 samples, a stigmatic focusing 90° magnetic sector field for high dispersion and high transmission and a linear (4 ppm) ion detection device. The complete measuring procedure and data evaluation of up to 13 samples in a sequence is performed automatically, without breaking the vacuum, under computer control. The software is modular. Each software module controls a defined instrument function and can be adapted to each particular measuring problem by calling the appropriate set of parameters from store. Together with the high sensitivity of the mass spectromater the automatic measuring procedure leads to reproducibilities of the ratio measurements in the range of $10^{-5}$.

*Varian AG, Steinhauserstrasse, 6300 Zug, Switzerland.*

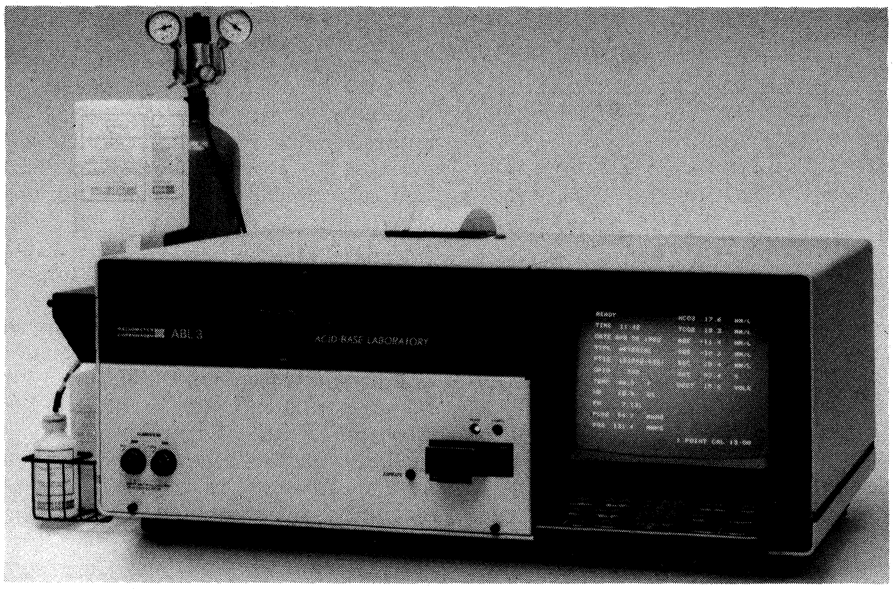

*Radiometer's ABL3 Acid-base laboratory*

### Fraction collector

The LKB 2111 MultiRac fraction collector is based on the moving head principle. Rather than position the vessel under the drop head, the latter positions above the vessel, the height being adjusted manually so that most types of receiving vessel can be accommodated, with an adaptor for large vessels. Transverse movement is controlled electronically and the rack size may be programmed prior to a run. The MultiRac accepts most buffers and organic solvents. All parts coming into contact with the solvent are of polypropylene or Ryton. Volumes vary from 10 μl to 3.5 litres and 295 litres can be handled on one run. The MultiRac has a choice of three collection modes - in time, in drop numbers and by pump control when connected to the LKB VarioPerpex pump. It is safe to operate at temperatures of 0 - 40°C. The basic LKB MultiRac fraction collector includes a shelf kit, a level sensor, a self-diagnosis adaptor, drop counter, tube racks and rack trays, dust cover and waste trough.

*LKB Instruments Ltd, 232 Addington Road, South Croydon, Surrey CR2 8YD UK.*

### Computer control for TLC

The Camag TLC/HPTLC scanner can now be controlled by a computer, a desk-top model in the HP 9800 series, which takes charge of all functions and all data processing operations. The software supplied with it, which has been specially developed for the needs of TLC scanning, permits automatic location of scanning tracks, evaluation by peak height or peak area, calculation of linear or non-linear calibration functions, calculation of fraction quantities, print out of full analysis report, dialogue programming and storage of scanned parameters in routine analyses. This computer control minimises the number of manipulations required in routine analysis: plate insertion, call-up of stored scanning programme and withdrawal of complete analysis report. It also ensures precision and accuracy of analyses as variations in distance between samples, and slight skew of the chromatogram tracks are compensated by automatic peak centering.

*Camag, Chemie-Erzeugnisse and Adsorptions-technik AG, 4132 Muttenz/ Schweiz, Homburgerstrasse 24.*

### Ternary liquid chromatograph

The Varian model 5060 is a new addition to the 5000 series of liquid chromatographs. It is built around a processor-controlled single piston pump. Ternary solvent system capability is standard and a number of new options, including a new injection valve, a variable wavelenght uv detector, and a software option allowing two-way serial communication with data system, are available.

*Varian AG, Steinhauserstrasse, 6300 Zug, Switzerland.*

### Recorder IEEE interface

An IEEE interface for the Store range of magnetic tape instrumentation recorders is announced by Racal Recorders. The result of an intensive development programme, the Model 110 interface is both a listener and a talker device which integrates any machine in the Store range into a computer controlled instrumentation system. Receiving computer instructions through the interface, the recorder can respond to mode and function commands and also supply status and positional data into the GPIB (general purpose interface bus) for use by the system controller.

*Racal Recorders Ltd, Hardley Industrial Estate, Hythe, Southampton, Hampshire, SO4 6ZH, UK.*

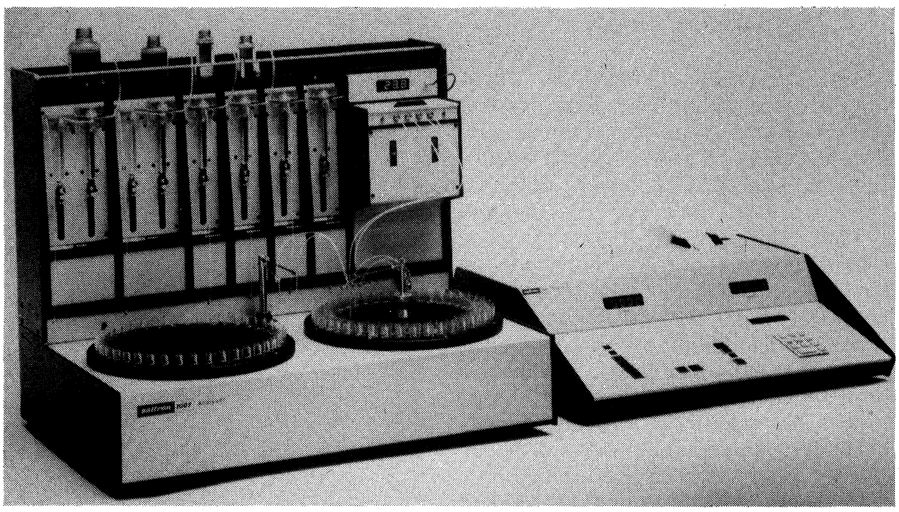

*The Saitron 9601 dual channel automatic analyser*

### Peristaltic pump

LKB Instruments has produced the LKB 2132 MicroPerpex peristaltic pump. By pumping a known volume through the pump a wide range of calibrated flow rates is obtained. Pump parameters can be continuously monitored through the LED display. These parameters include flow rate, on/off, rotational direction and calibration status. The microprocessor controlled instrument processes and stores information from the calibration and a uniform motor speed is ensured by the quartz crystal which regulates the microprocessor signal frequency. The MicroPerpex features a new roller system which consists of ten peripheral rollers and one central spring roller which is claimed to make it possible to maintain a constant optimal pressure on the tubing. The pump is supplied with two pump heads, each with a flow range of 0.5 - 500 ml/h. These heads may be operated separately or in parallel to achieve a maximum flow rate of 1000 ml/h.

*LKB Instruments Ltd, 232 Addington Road, Selsdon, South Croydon, Surrey, CR2 8YD, UK.*

### Dual channel automatic analyser

One of the latest additions to the Saitron range of laboratory instruments is the 9601 dual channel automatic analyser available in the UK from Arnold Horwell. The unit consists of a microprocessor-controlled dual channel system which can dispense up to three different reagents in the sample preparation stage, enabling it to carry out most types of colorimetric and kinetic enzymatic analysis. The preparation unit has two 80-place sample plates with a variable ratio diluter and two dispensers for each. Incubation temperatures of 25, 30, and 37°C are available, and incubation times can be set between 20 seconds and 60 minutes, providing a maximum sample throughput of 180 samples per channel. The photometric unit consists of two automatic flow-through cells with optical paths of 10mm. The light source is a tungsten filament lamp providing a spectral range of 340 - 700 nm. The temperature of the cells is controlled by forced fluid circulation using a Peltier effect heating/cooling system. The programming-printer unit provides control for the analytical processes. It has a memory storage capacity of up to 20 test procedures which can be stored indefinitely. Once a programme has been selected the computer prints out the various parameters of the tests eg, incubation time, sample and reagent volumes, wavelength etc. When the results are printed out, normal values for the particular test are also shown to alert the operator to an abnormal result.

*Arnold R Horwell, 2 Grangeway, Kilburn High Road, London NW6 2BP, UK.*

### Automated electrophoresis system

Gelman Sciences announce the introduction of the Autophor 128, for high volume testing laboratories in government, research, clinical and industrial environments. This automated electrophoresis system can run up to 128 patient samples in 3½ hours. The densitometer module is Gelman's ACD-18, which offers many capabilities as a stand-alone unit for scanning CK's, LDH's and other samples. The ACD-18 computes relative percent and gram value, as it plots the electrophoresis pattern of each sample. Serum protein results reported include total concentration and computed values for individual fractions (relative percent and concentration), and A/G ratio. Of special value in clinical testing is the standardisation of test conditions with the Autophor 128 - pH and ionic buffer strength, sample application and charge characteristics of the membrane, and the electric field applied to the sample are all controlled automatically. Results are shown on LED readouts and printed graphs on charts. Sample size is 25 microlitres, stain is Gelman's Ponceau S, rinse is 10% acetic acid and clearing is done with Gelman's Super Sepra Clear. An alarm is audible for low liquid levels in membrane rinse reservoirs, which prevents loss of the run or patient samples. The electrophoresis tank features a high voltage safety interlock and solidstate buffer cooling. The applicator has eight capillary tips. The membrane is cellulosic on Mylar backing in a continuous roll for up to 128 samples per roll.

*Gelman Sciences Ltd, 10 Harrowden Road, Brackmills, Northampton NN4 OEB, UK.*

### Microcomputer

Hallamshire Technical Services have announced a microcomputer specifically designed for connection to scintillation counters, TLC scanners, HPLC detectors etc – the Micromat IN 5000. Standard software has been developed and is supplied with the unit. Programming is in a language which is a development of BASIC. The language incorporates input/output instructions available in languages such as FORTRAN. The computer has been designed to facilitate proper interfacing – 'hand shaking'. The unit features the facility for a large buffered input which can be in a variety of forms, digital, analogue, single or multiple. The computer can control devices by use of programmed instruction and is impervious to interruptions in the mains supply.

*Hallamshire Technical Services, 11 Union Road, Sheffield S11 9EF, UK.*

### Computing integrator

Pye Unicam's CDP4 computing integrator is a microprocessor based unit which incorporates permanently stored programs to handle most common chromatographic analyses. Six standard data reduction procedures can be completed and these can be supplemented with features such as peak height, multi-level calibration, and statistics. Entries are shown on an eight-character LED display to permit verification before acceptance. Up to four complete method files can be stored in the RAM memory of the CDP4. Each contains information necessary to collect and reduce the data for a given analysis, and may also include a sample file with multiple samples. The CDP4 is a self-contained unit which can be used with most chromatographic systems. It is equipped with six programmable switch closures to control external devices such as autosamplers, valves or alarms. Addition of a cassette recorder enables the internal RAM memory to be extended for storage of further peaks and parameter files.

*Pye Unicam Ltd, York Street, Cambridge CB1 2PX. UK.*

### Microprocessor controller for LC

The KLIC 1 from Kratos, is a microprocessor-based controller used for automating liquid chromatographs. It uses a coded function keyboard and common chromatography language rather than computer language.

Complete programmes can be stored on tape using a built-in tape deck. After the taped programme is 'run into memory', the programme can be modified in memory without altering the programme as it is recorded on tape. Capabilities of the KLIC 1 include gradient and flow programming, wavelength control on variable wavelength detectors, plus programmable control overup to 40 on/off external devices such as autosamplers, fraction collectors, solenoid valves and solvent switching valves. The interactive feedback design permits continous monitoring of various system components. A large readout panel includes LED displays for many system operating conditions and programme instructions. All parameters are continuously displayed. An optional alphanumeric printer provides a permanent record of the KLIC 1's operation during an unattended run.

*Kratos Ltd, Barton Dock Road, Urmston, Manchester M31 2LD, UK.*

### Trace organics concentrator

Valco Instruments have introduced a new system for the concentration of trace organics to levels detectable by gas chromatography and gc/mass spectrometry. It is called the automatic trace organics concentrator, model ATOC-1. Based on the sparge/purge-trap-rapid desorption technique it allows analysis of trace organic pollutants. Composed of Valco switching valves, temperature controllers and sequence programmers for gas chromatography, the automatic trace organics concentrator allows the user to automate this and other related concentration procedures without compromise of the goals defined by the sparge/purge-trap-rapid desorption technique. It features a separately heated valve enclosure and heated transfer lines, traps which dismount for on-site sampling, and panel mounted digital settings which are front panel adjustable.

*Valco Instruments Company Inc, P O Box 55603, Houston, Texas, USA.*

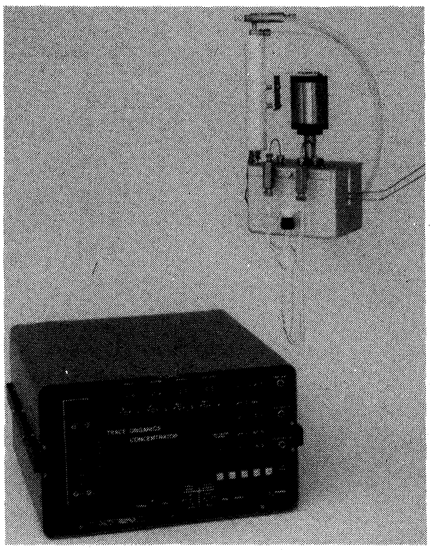

*Valco's trace organics concentrator*

*KLIC 1 controller from Kratos*

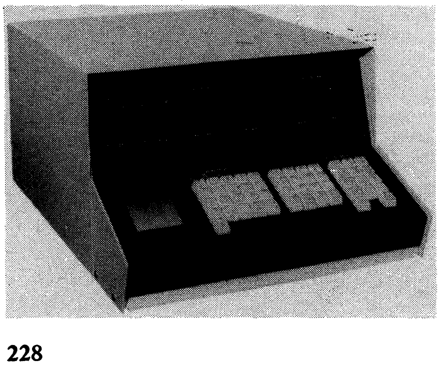

### 16/32-bit development system

Celdis have a new 16/32-bit multiple station development system known as the EXORmacs. The basic system comprises a microcomputer chasis with slots for up to 15 printed circuit boards, an M6800 based CRT, an EXORdisc III 1M-byte dual floppy disc drive unit and a 132 column, 180 cps printer. The chassis houses a power supply, cooling fans and front panel controls. Five boards are included in the basic system. These are a M68000 processor module, a debug module with bus arbiter and programmable timers, an M6801 based floppy disc controller board, a 128K-byte dynamic random access memory (RAM) board and a 32K-byte static RAM board. All boards include self test firmware. The system allows for up to two users but for applications requiring multi-user capabilities this can be expanded by adding a hard disc controller and a hard disc drive, multiple channel communication modules, and up to 8 additional peripheral terminals as required.

*Celdis, 37 Loverock Road, Reading, Berks RG3 1ED, UK.*

### Thermal analyser

The Mettler 2000C is a thermal analyser capable of carrying out simultaneous differential scanning calorimetry and thermogravimetry up to $1200^{\circ}$C. The top loading all alumina furnace can be loaded at high temperature and is corrosion resistant to most atmospheres; for ultimate corrosion resistance there is a special furnace. The built-in vacuum and gas flow system offers atmosphere control and there is also an accessory furnace which allows operation up to $1600^{\circ}$C. The balance is controlled with push-button tare and has the ability to detect weight changes of $10\,\mu g$ for sample weights up to several grammes.

*MSE Scientific Instruments, Manor Royal, Crawley, Sussex. UK.*

### Hard copy record

VT100 users can now obtain a printer port option (VT1XX) from Rapid Recall which allows an entire screen's contents, specified sections of a screen or incoming data, to be printed at will. Available communication rates range from 150 to 9600 baud. All screen-to-printer operations are implemented using keyboard commands. The VT100 is also available from Rapid Recall. It is a compact video terminal which offers a wide range of features as standard. All functions such as baud rate, character size, screen format, tabs etc, are set using the keyboard and are stored in non-volatile random access memory within the unit. Characters are presented on the VT100 in a 7 x 9 dot matrix character font. Also available are double-width and double-height characters that may be selected on a line-by-line basis. Normal and reverse video are available. The VT100 can display 132 characters per line, with a maximum of 24 lines per page. A separate numeric/function keypad can be used to initiate software dictated sequences at a single keystroke, or for rapid numeric data entry.

*Rapid Recall Ltd, Rapid House, Denmark Street, High Wycombe, Bucks HP11 2ER, UK.*

### TLC spot application

The Camag Nanomat available from Baird & Tatlock enables spot application of quantitative and qualitative samples in TLC, especially in HPTLC. The apparatus is suitable for linear TLC plates and for circular chromatograms. The transfer of the sample is made by capillary pipette, eg, glass capillary for normal TLC layers, application capillary with Pt-Ir tip or fixed volume dosage pipette for HPTLC plates. The capillary pipette is filled externally from the apparatus and placed in the opening of the magnetic head where it is held above the layer. A release button is pressed and lowers the capillary on to the TLC plate and discharges its contents. It then returns automatically to its original position and is removed. Speed of lowering and delay time on the layer are controllable. With correct adjustment damage to the layer is prevented. Positioning notches at 5mm intervals for linear application and at $15^{\circ}$ on the turntable for circular application facilitate the operation. The precision of the application is claimed to be very high. A100 ml fixed volume dosage pipette produces an application spot of approximately 1 mm diameter and the centre of the spot is contacted every time when repeated solvent applications are made.

*Baird & Tatlock (London) Ltd, P O Box 1, Romford, RM1 1HA, UK.*

### Microelisa reader

For laboratories requiring automatic facilities when reading Microelisa test results, Dynatech Laboratories has introduced a new Auto Reader - the MR580. It is claimed that using this instrument all 96 wells on a Microelisa plate can be read and the results printed out in one minute. A dual wavelength feature eliminates the effects of scratching and fingerprints on the plates. The sample and reference filter enables preselection of wavelengths at 410, 450, 490 and 570 nm. Overall wavelength range is from 200 nm to 600 nm. A built-in alpha-numeric printer recorder will identify and display the results in absorbance units to three decimal places. The MR580 is essentially a single channel photometer designed to vertically measure the light absorbance of a sample in the Microelisa or Microtiter plate well. Conversion to single wavelength system is also possible. A blanking matrix offers automatic blanking on a well A-1 of a Microelisa plate or manual selection for blanking on any well or group of wells.
*Dynatech Laboratories Ltd., Daux Road, Billingshurst, Sussex RH14 9SJ, UK.*

### Electronic programming systems

Control and Readout announce electronic programming systems type 802 and 803. The 802 model allows a process, usually temperature or pressure, to ramp at a predetermined rate to any desired level and hold for the required time. All settings are made digitally to ensure accuracy and repeatability. The 803 model provides up to 15 stages of ramp and dwell for more complex applications. A microprocessor is employed and an important feature is that the operator can see the stage reached in any programme via LED indicators - these also show whether the system is in ramp or dwell.
*Control and Readout Ltd, Woods Way, Goring-by-Sea, Worthing, West Sussex, BN12 4TH, UK.*

### FFT analyser

B & K Laboratories have just announced a new FFT signal analyser which can be used to widen the scope of signal analysis. The Type 2033 high resolution signal analyser as it is known provides all the basic measurement facilities required for conventional FFT analysis but has the addition of an instant 'zoom' feature which enables resolution of any part of the spectrum being examined to be increased ten fold wherever necessary and as often as required without destroying the original time function. This is achieved by means of a large input memory which in combination with a flexible trigger enables the 2033 to analyse both continuous and transient data in conjunction with its conventional Fourier analysis system. Thus

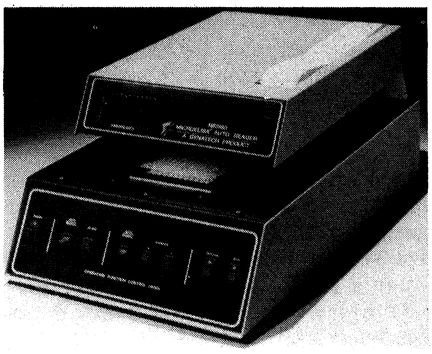

*Microelisa reader from Dynatech*

it can operate in two modes: as a 400 line FFT analyser sampling the input and transforming 1k samples at a time into the frequency domain and so presenting a constant bandwidth spectrum at 400 equally spaced frequency intervals across a frequency range of 0 to 20 kHz; and as a high resolution analyser operating on 10k samples of the input, ie a ten times longer time function. In this way any selected tenth part of the frequency spectrum chosen can be 'zoomed' to fill the entire display screen with the 400 line frequency intervals, thus making possible a 4000 line frequency analysis of the total spectrum. The most important point about this is the repeatable 'zooming' without recording a new time function. The 2033 can also apply 'scan' analysis. In this mode it is possible to extend the time of a transient signal so that it can be analysed in slow motion. The 2033 has several other features which makes it suitable for use in the analysis of acoustic and vibration signals, shocks and transients, machine run-ups, vibration of cyclic machinery, order analysis etc. and it can be connected to IEC 625/IEEE Std. 488 interface bus.
*B & K Laboratories Ltd, Cross Lances Road, Hounslow, Middlesex, UK.*

### Digital cassette recorder

B & K Laboratories announce the release of a new digital cassette recorder. This recorder Type 7400 as it is known offers a solution to the problem of recording digital outputs from measuring instruments. Up to 500k-Bytes of data, transmitted over the IEC/IEEE or B & K low power interface bus, can be recorded on a standard digital tape cassette and reconstructed on the bus later. The 7400 incorporates manual and remote control, and the recording formats used meet ECMA 34 and ECMA 41 (basic system). As the recorder can be manually as well as remotely controlled, it can be used independently of an IEC/IEEE controller in recording or reading data in the field. A 4-digit tape location display indicates the position of the tape and a search function can be used to speed the retrieval of recorded data. Here the location of the data is entered into the 7400, which then makes a search through the tape for the location. The data standing at that location may then be read over the interface bus, or new data recorded. Remote control of the 7400 is over its interface which is compatible with IEC 625-1 and IEEE Std. 488. Cassettes recorded on the 7400 can be read by ECMA compatible computer terminals, and vice versa. The recorder can record or read data at 1000 bytes/second (average) with a tape speed of 15 ips. Error checking procedures are incorporated. The search speed is 30 ips and the rewind speed is approximately 100 ips. The 7400 recorder can be powered from an AC or DC source.
*B & K Laboratories Ltd, Cross Lances Road, Hounslow, Middlesex, UK.*

### Data acquisition system

Pye Unicam have introduced the Philips PM 4001, a universal data acquisition system for scientific and industrial applications. This system is based on the PM 4000 compact data logger with additional hardware and software. The system can handle up to 950 analogue and/or digital inputs using IEC-625 instrument bus and parallel interfaces as well as a combined V24 (RS232C)/20 mA interface card. Thermocouple and Pt100 resistance thermometer linearisation is standard and strain gauge measurements can be performed. The PM 4001 is a completely modular system. The mainframe holds 50 input channels while satellite input units hold 100 channels each. Data processing facilities allow conversion from imput voltages or currents to various physical values following the formula M = ax + b, where a and b are constants. Physical units can be added on outputs. Calculations of mean values and sums of the different analogue and digital inputs are also possible. An ouput buffer allows storage of values that cannot be fed directly to a peripheral unit.
*Pye Unicam Ltd, York Street, Cambridge CB1 2PX, UK.*

### In-sync multi-channel traces

All standard multi-channel hardcopy recorders with three or more channels normally provided out-of-sync traces, making it difficult to analyse results of mass chemical analyses, especially when the required signal has a small amplitude and high noise level, and when all traces are fully overlapping. With the Bryans Series BS314 or BS316 mainframe factory-fitted with the DataSyn Unit all the traces are in time-synchronisation. An optional feature available with this unit is an IEEE-488 1978 interface, which allows the recorder to be operated via the standard IEEE and IEC interface bus commands. These interfaces are compatible with talk and listen modes.
*Bryan Southern Instruments Ltd, Willow Lane, Mitcham, Surrey, CR4 4UL, UK.*

### Photoacoustic spectrometer

EDT's photoacoustic spectrometer, model OAS 400, is now available with an IEEE 488 computer interface to which can be connected directly a wide variety of minicomputers and microcomputers. This allows the instrument to be controlled by the computer and for data processing and manipulation to take place inside the computer, prior to the completed spectrum being displayed to the integral xy recorder for hard copy storage.

A range of software programs for the Commodore PET microcomputer have been developed by EDT. These allow the user to acquire up to four spectra as well as a carbon black reference spectrum for subsequent manipulation and processing. Further spectra can be stored on a floppy disc which is an optional extra with the system. Two spectra can be added, subtracted or given as a ratio allowing baseline subtraction and normalisation of lamp intensity. User specific programmes can be developed using the optional BASIC compiler.

The program in standard form is designed to run on a 32K PET with cassette program storage. An alternative version designed for use with the Comp-u-Think diskette is also available.

*EDT Research, 14 Trading Estate Road, London NW10 7LU, UK.*

### Liquid scintillation counter

Microprocessor control and automatic analysis of up to 300 samples per run are features of Beckman's LS-7500 liquid scintillation counter. The counter has a built-in library of ten standard counting programs that satisfy the most frequently requested liquid scintillation applications. Command tower programming gives the operator the versatility to edit counting parameters.

Command towers, inserted in the conveyor belt preceding a group of samples, select any one of the 10 programs. The user sets a command tower and pushes the Auto button to activate the program. Time and error of the program is edited by dialling a change on the second command tower. A program summary is printed automatically to confirm the desired counting parameters. Up to 300 samples can then run unattended. Instrument calibration and quench monitoring by H number are also performed automatically.

*Beckman-RIIC Ltd, Turnpike Road, Cressex Industrial Estate, High Wycombe, Bucks HP12 3NR, UK.*

### Calendar

**Editor's Note:**
*Organisers of conferences, seminars etc. should send details for inclusion in this calendar as soon as the relevant information is available and not later than three months before the event.*

**Microcomputer applications in health care**
December 5, London.
*Dr J.P. Woodcock, Department of Medical Physics, Bristol General Hospital, Guinea Street, Bristol.*

**Atomic absorption and emission spectrometry**
December 8-12, Loughborough.
*Dr J.F. Tyson, Department of Chemistry, Loughborough University of Technology, Loughborough, LE11 3TU.*

**Salon du Laboratoire**
December 8-13, Paris.
*Commissariat General, 40 rue de Colisee, 75381 Paris cedex 08, France.*

**Laboratory methods of handling and analysing toxic substances**
December 9, London
*The Secretary, Analytical Division, The Chemical Society, Burlington House, London W1.*

**Microprocessors and microcomputers, interfacing and applications**
December 14-19, Tallahassee, Florida
*American Chemical Society, 1155 Sixteenth Street, N.W., Washington DC 20036, USA.*

**1981**

**London Laboratory Conference**
February 24-26, London.
*A.J. Holdsworth, Manager, Exhibition & Conference Group, Morgan Grampian (Process Press) Ltd, 30 Calderwood St, London SE18.*

**The use of chemical nomenclature**
March 24-26, London.
*The Symposium Organiser, Laboratory of the Government Chemist, Cornwall House, Stamford St, London SE1.*

**Laboratory '81**
April 1-2, Glasgow
*Curtis Steadman, 34-36 High Street, Saffron Waldon, Essex.*

**Biotechnology — second European Congress**
April 5-10, Eastbourne
*Secretariat, ECB2, c/o Society of Chemical Industry, 14 Belgrave Sq, London SW1 8PS. UK.*

**International symposium on electroanalysis in clinical, environmental and pharmaceutical chemistry.**
April 13-16, Cardiff.
*Short Courses Section (Electroanalysis Symposium), UWIST' Cardiff CF1 3NU.*

**Anglo-Dutch symposium on quantitative organic analysis**
April 23-24, Noordwijkerhout, Holland
*Miss P.E. Hutchinson, Royal Society of Chemistry, Analytical Division, Burlington House, London W1.*

**Association of Official Analytical Chemists, 6th Annual Spring Workshop**
May 12-14, Ottawa.
*Donald L. Grant, Health Protection Branch, Health and Welfare Canada, Tunney's Pasture, Ottawa, Ontario KIA 0L2, Canada.*

**Automatic chemical analysis summer school**
July 5-10 Swansea.
*Professor D. Betteridge, Department of Chemistry, University College of Swansea.*

**Euroanalysis IV**
August 23-28, Helsinki, Finland.
*Association of Finnish Chemical Societies, Executive Secretary, Pohj, Hesperiankatu 3B10, SF-oo260 Helsinki 26, Finland.*

**1982**

**12th Annual Symposium on the Analytical Chemistry of Pollutants**
April 14-16, Amsterdam.
*Prof Dr R.W. Frei, Congress Office, 12th Annual Symposium on the Analytical Chemistry of Pollutants, Congress Bureau, Vrije Universiteit, PO Box 7161, 1007 MC Amsterdam, The Netherlands.*

**International Congress on Automation in the Clinical Laboratory**
April 19-22, Barcelona, Spain.
*Dr. R. Galimany, Departmento de Analisis Clinicos, Seccion de Automatizacion, Cuidad Sanitaria 'Principes de Espana', Hospitalet de Llobrogat, Barcelona, Spain.*

